# Understanding the Dynamics of consumer behaviour and purchase intentions for Green Cosmetic Products in North India: A Gender-Neutral Approach

**DOI:** 10.12688/f1000research.151629.1

**Published:** 2024-06-10

**Authors:** Neha Singh, Babita Rawat, Dhani Shanker Chaubey, Ambica Prakash Mani, Aditi Singh

**Affiliations:** 1 Management, Uttaranchal University, Dehradun, Uttarakhand, 248007, India; 2 Management, Graphic Era Deemed to be University, Dehradun, Uttarakhand, 248001, India; 3 Management, Teerthanker Mahaveer University, Moradabad, Uttar Pradesh, 244001, India

**Keywords:** Green cosmetic products, Purchase behavior, Purchase intention, TPB, organic skin care, multi-group analysis, Environment Concern

## Abstract

**Background:**

The increasing popularity of green cosmetic products among consumers who prioritize skin health, quality, and beauty has driven the production of these products. Growing awareness of harmful toxins in traditional cosmetics is leading consumers to prefer natural alternatives. This study aimed to identify the factors influencing Indian consumers' buying behaviour and purchase intentions toward green cosmetics, focusing on both male and female consumers.

**Methods:**

To achieve this, a thorough evaluation of the literature from Scopus-indexed journals was conducted, employing the theory of planned behavior as the theoretical framework. The study used Smart PLS 3.0’s structural equation modeling to analyze data and performed a multi-group analysis to explore variations in purchasing intentions and actions between male and female consumers.

**Results:**

The research indicated an increasing demand for green cosmetics among males in North India. Findings revealed that subjective norms significantly influenced both purchase behaviour and purchase intention. Additionally, perceived behavioural control and subjective norms positively impacted purchase intention, which in turn indirectly affected purchase behaviour. Interestingly, environmental concerns did not influence consumers' actual purchasing intentions or behaviour. The analysis also demonstrated no significant differences between male and female consumers regarding the impact of various factors on their purchase intentions and behaviours.

**Conclusion:**

These findings provide valuable insights for policymakers and marketing managers aiming to promote green cosmetic product purchase behaviour. Recognizing that subjective norms and perceived behavioural control are crucial drivers of purchase intention and behaviour can help in devising effective marketing strategies. Despite the absence of significant gender differences in the factors influencing purchase behaviour, the increasing interest in green cosmetics, especially among males, highlights a broader market potential for these products. This research underscores the importance of focusing on social and behavioural influences to encourage the adoption of green cosmetics

## 1. Introduction

Over the last few decades, more people have been opting for natural and eco-friendly products as they change their habits when it comes to choosing everyday items. This trend extends to cosmetics, which a large portion of consumers use as part of their daily routine ( Yu & Lee, 2019). As of late, customers have become more conscious of their purchasing decisions regarding their health and the environment ( Chin *et al*., 2018). This has led to a preference for green cosmetic products ( Seinfeld & Pandis, 2016). Different terms have been used in previous studies to describe the concept of ‘green’, including “environmentally friendly”, “environmentally responsible activities”, "pro-environmental consumer behavior”, “pro-environmental consumption behaviour” and “sustainable” (S. H. Kim & Seock, 2019; Sadiq & Adil, 2021; Taufique *et al.*, 2017). Green products have been defined by ( Saxena & Khandelwal, 2008) as products that are durable, chemical free, and sometimes made from recycled materials, and minimally packaged, while ( Peattie, 2010) describes green products as having significantly improved environmental and social performance in production, use and disposal as compared to chemical or competitive product offered in the market. These cosmetics may include hair dye, lipstick, lotion, sunscreen, hair gel, shampoo, conditioner, toothpaste, and deodorant. Many people now consider these products to be essential items ( Sahota, 2014). As given in the *Drugs and Cosmetics Act of 1940* in India, a “cosmetic" is defined as any product that is meant to be applied or introduced to the human body, or any part of it, for cleansing, enhancing beauty, improving attractiveness, or changing appearance. This includes any item that is intended to be used as a component of a cosmetic. It appears that cosmetology was first developed in ancient Egypt and India. ( Patkar, 2008) The use of cosmetics dates to the Indus Valley civilization (2500-1500 B.C). In the past, cosmetics made through traditional methods included microplastics and other harmful chemicals that had detrimental effects on both people and the environment ( Andrady, 2011) **;**( Seinfeld & Pandis, 2016). However, consumers are now more conscious of how their choices impact their health and the planet. As a result, they are becoming more cautious and selective in their consumption pattern and purchase behaviour. Products like hair colour, lipsticks, colour cosmetics, face creams, deodorants, soaps, hand wash, body wash, dental care items, shampoos, UV filters, detergents, sunscreens, and fragrances can have harmful effects on the environment when washed off of people's bodies and into bodies of water like lakes, rivers, and oceans ( Klaschka, 2015).

Scholars have noted that green products can be effective in promoting environment safety throughout their complete life cycle ( Maniatis, 2016) and can help to reduce the damage caused to nature and Mother Earth ( Tomasin *et al*., 2013). To gain insight into consumer behaviour, it is crucial to understand why individuals purchase green cosmetics and how frequently they do so. It's important to keep in mind that just because someone is aware of environmental concerns and the impact of their cosmetic choices does not always mean they will purchase green products.

Research has indicated that consumer views on green marketing and consumption vary; some may have favourable intentions and perceptions, while others may have bad experiences and favourable conventional methods of consumption ( Lim *et al.,* 2013). Companies must comprehend these divergent viewpoints to sell their green products properly. Therefore, to boost demand for eco-friendly cosmetics, it is imperative to research several aspects impacting consumers' purchase intentions. ( Paul *et al*., 2016; Hsu *et al.*, 2017) authors also presented in their studies that the successful development of green products is crucial for business growth with eco-friendly strategies. There is a growing trend in India, where men increasingly use cosmetics for various purposes. Both male and female consumers in India choose eco-friendly and natural cosmetic products, resulting in a sales increase of around USD 1.2 billion in 2023. While past studies have mostly focused on female consumers and their purchasing behaviour, the current market scenario demands a study on men's cosmetic usage and their preference for natural cosmetics. There is a growing trend of guys using makeup for various objectives in India. In India, eco-friendly and natural cosmetics are preferred by both male and female consumers, leading to a projected 1.2 billion USD increase in sales by 2023. Previous research has predominantly examined female consumers and their purchase behaviour; however, given the state of the market, a study must be conducted on the cosmetic consumption of males and their inclination towards natural cosmetics ( Amberg & Fogarassy, 2019).

This research paper delves into how customers view green-beauty items' worth and the elements that sway their buying choices. The investigation employs a method with a survey to understand consumer actions and motives regarding green cosmetics. It also examines how environmental and health worries affect the connection between the desire to purchase eco cosmetics and the factors that lead to consumer reluctance. The study helps marketers and producers of green cosmetic products manufacture, distribute, and advertise the advantages of green cosmetics to customers by providing an understanding of this issue. Furthermore, the study divides the cosmetics business into gender-specific divisions and offers separate findings for customers who identify as male or female.

## 2. Literature review

Behaviors are shaped by our intentions, which are developed based on our attitudes how we perceive norms, and the level of control we believe we have over our actions ( Ajzen, 2002). *Attitude* refers to a person's evaluation of behaviour, while subjective norms involve social pressures that support or discourage behaviour, *perceived behavioural control* reflects how easy or difficult it is to perform a behaviour based on past experiences and anticipated obstacles and *Purchase intention* ultimately leads to actual purchasing behaviour, with environmentally conscious consumers willing to pay a premium for natural products ( Ajzen, 1991). Purchase behaviour refers to the consumer's decision-making process and evaluation of products or services ( Gera & Jain, 2020) **.** Although there has been a lot of research on the topic, prior studies have not explored the precise reasons that underlie the attitude-behavior gap for green cosmetic goods ( Kumar *et al*., 2019). To comprehend customer behavior and buying intentions, the TPB model is frequently employed. In this study, TPB was used to analyze the purchasing behaviour of men and women toward natural cosmetics ( Sparks *et al.*, 1992).

### 2.1 Product Knowledge (PKN)

When shopping for goods, customers tend to compare factors such as quality, price, and utility before making a purchase decision ( Peattie, 2010). This is especially true for shopping for goods, which requires more effort from consumers to seek information and understand the product. Green cosmetic products come under the category of these goods. This rational approach to product information means that consumers are less likely to refuse to buy green products based solely on their attitudes ( Quester & Lin Lim, 2003). However, convenience goods, which are low-value and frequently purchased, may be subject to attitude bias or brand loyalty when it comes to choosing green alternatives. Researchers have studied the influence of product knowledge regarding green cosmetic products on environmental concerns to understand consumer purchase behaviour. The hypothesis is set accordingly, and a multi-group analysis is done among male and female consumers.

H1: Consumers' product knowledge and its influence on environmental concern is gender neutral.

### 2.2. Perceived Behavioural Control (PBC)

When it comes to personal care ( Gadema & Oglethorpe, 2011) and cosmetic products ( Richter, 2010), many consumers prioritize green and environmentally-friendly options. However, implementing a green purchasing lifestyle can be difficult. One factor affecting consumer behaviour is perceived behavioural control (PBC), which refers to the belief that one has the necessary resources and sufficient opportunities to positively carry out a desired purchase behaviour ( Ajzen, 1991). PBC emphasizes situational constraints, similar to self-efficacy and barriers ( Ajzen, 2002). Unfortunately, many studies do not specify the constraints or barriers that hinder consumers from making green choices. Instead, respondents are often asked direct questions regarding the ease or difficulty of performing a certain action, leaving room for interpretation. In this context, we believe that perceived behavioral control is closely related to consumer purchasing behaviour. Therefore, we suggest hypotheses 3 and 4.

H2: Perceived Behavioural Control (PBC) and its influence on purchase behaviour is gender neutral

### 2.3. Environment Concerns (EC)

The growing concern for the environment has led to a rise in demand for green cosmetics. These products are preferred for their natural ingredients, green buying behaviour, and positive impact on the environment ( Bamberg, 2003). Researchers argue that the consumers who prioritise green cosmetics are conscious of environmental issues and seek to understand the origin of raw materials used in the products they purchase ( Balderjahn, 1988; Chenery, 2004; Oyewole, 2001). Consumers opt for those cosmetic products that do not contribute to pollution and have fewer carbon footprints ( Bamberg, 2003). The ingredients of a cosmetic product play a significant role in determining its popularity among environmentally aware consumers; this trend is commonly referred to as environmental awareness (S. Kim & Seock, 2009).

H3: Consumer Environmental concern and its influence on purchase intention is gender neutral

### 2.4. Purchase Intentions (PI)

Purchase intentions refer to an individual consumer’s willingness to be involved in buying behaviour, which reflects their concern for the product and its quality. This is considered to be a significant factor that influences their behaviour. Many researchers say purchase intentions for green cosmetic products are affected by consumers' likelihood of purchasing other green cosmetic products ( Chen & Chang, 2012). On the other hand, he argues that it reflects the consumer's desire to protect or preserve the environment by purchasing green products ( Rojšek, 2001). ( Fraj & Martinez, 2007) suggests that green cosmetic product purchase intentions can be measured by considering purchasing green products, switching to green brands, and choosing a green product version. However, researchers have not reached a consensus on how product knowledge affects green purchase intentions.

H4: Consumer purchase intention and its effect on purchase behaviour are gender neutral

### 2.5. Subjective Norms (SN)

Subjective norm is the pressure that an individual consumer perceives from society to perform a specific behaviour. When consumers believe that others highly regard organic skin care products, they are more likely to intend to purchase these items. Research has demonstrated that subjective norm significantly impacts the intention to manage skin effectively ( Hillhouse *et al*., 2000). Moreover, studies on green consumer behaviour have revealed a strong correlation between subjective norms and the purchase intention of consumers ( Bamberg, 2003; Kalafatis *et al*., 1999).

H5: Subjective norm and its influence on purchase intention of cosmetics are gender neutral

**Theoretical framework**

Using a gender-neutral approach, a comprehensive theoretical framework is required to understand the dynamics of consumer behaviour and purchase intents for green cosmetic items in North India. The Theory of Planned Behavior ( Ajzen, 1991) serves as the foundation for this study, which looks into how people's intentions to buy green cosmetics are shaped by their views toward the products, social factors, and perception of their control over the purchasing process. The theory behind this research is that attitudes, subjective norms, and perceived behavioral control influence intentions and subsequent behavior. This study tries to examine how gender-neutral marketing and messaging techniques may lessen gender biases in the context of green cosmetics use. This is achieved by incorporating insights from the Gender Schema Theory ( Fornell & Larcker, 1981), which clarifies how societal gender norms and schemas influence individuals' perceptions and behaviors. Moreover, in light of the increasing significance of sustainability and environmental awareness in consumer choice, ideas from the literature on green marketing ( Peattie, 2010) and will be utilized to analyze how eco-friendly or green attributes, labelling, and messaging influence the consumers' perceptions and preferences for green cosmetic products. This theoretical framework integrates psychological, sociological, and marketing perspectives to offer a holistic understanding of the factors driving consumer behaviour and purchase intentions for green cosmetics in North India while accounting for gender-neutral considerations.

## Methods

**Figure 1.**
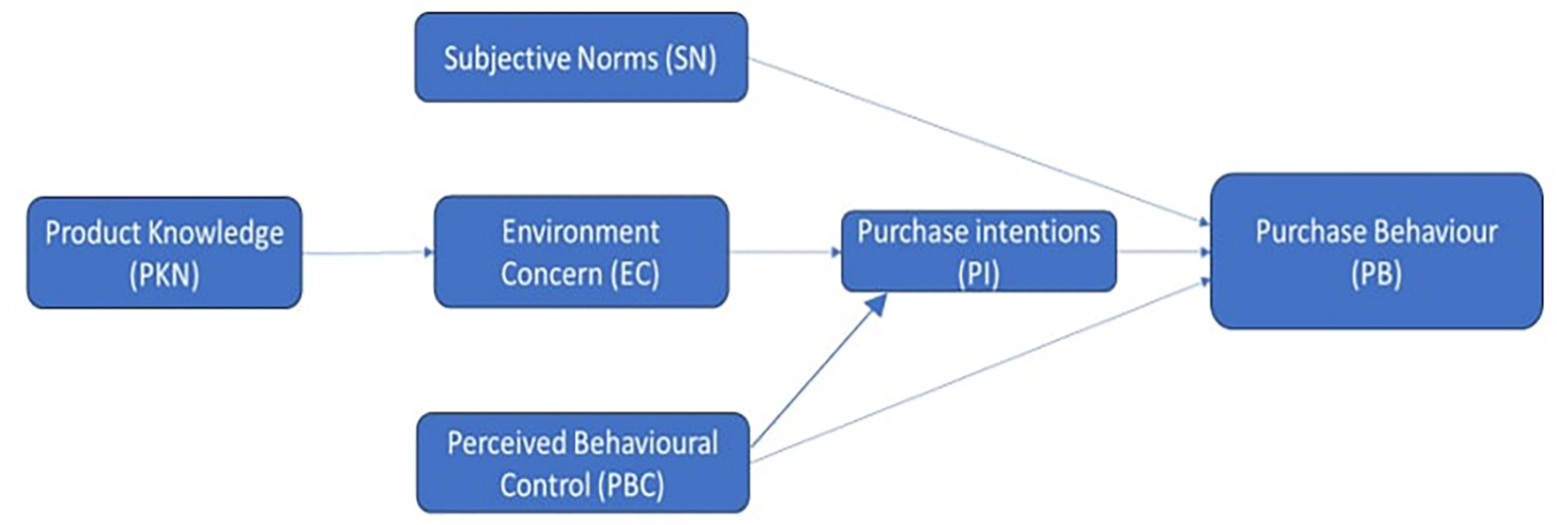
The conceptual Model based on TPB. **Source:** Author’s Data analysis.

This study utilised primary and secondary data with a descriptive research design to investigate factors influencing the purchase behaviour and intentions of male and female consumers in India towards green cosmetic products. Figure 1 displays the research objectives and methodology. A self-administered questionnaire with a five-point Likert scale and 29 items were distributed among consumers in Delhi, Dehradun, Faridabad, Sonipat, and Meerut in Oct 2023. Table 1 shows the studies supporting the factors selected for the study, and further measurement variables were developed. Independent variables included environmental concern, product knowledge, perceived behavioural control, and subjective norms; dependent variables were purchase intention and purchase behaviour. The Reliability test was conducted with the help of SPSS software and found to be 0.950, well above the threshold of 0.70. After assuring the reliability, a full-scale survey was carried out. Research has received 545 responses. After editing, 505 responses were found fit, leaving 40 insincerity-filled responses. Respondents' demographics are detailed in Table 2.

**Table 1.** Source Identification of Variables.

S.no	Related Factors	Supported studies
1	Environment Concern	( Aagerup & Nilsson, 2016; Al Mamun *et al*., 2018; Maichum *et al.*, 2016)
2	Availability	( Dhanwani *et al*., 2020; Kaufmann *et al*., 2012; Singh *et al.*, 2019)
3	Subjective Norms	( Barua & Islam, 2011; Bijauliya *et al.*, 2017; Boon *et al*., 2020; Mancha & Yoder, 2015; Wu & Chen, 2014)
4	Purpose of using the product	( Novak *et al*., 2003; Peterman, 1997; Xia & Monroe, 2009; Zaichkowsky, 1985)
5	Price Sensitivity	( Ali *et al*., 2010; Gabor & Granger, 1961; Rahim, 2016; Tellis, 1988)

**Source:** Author’s compilation.

**Table 2.** Construct Statistics.

Construct	Items	Factor Loading	Cronbach Alpha	CR	AVE
**Environment Concern**	EC1 EC2 EC3 EC4 EC5	0.802 0.835 0.774 0.861 0.856	0.883	0.915	0.683
**Purchase Behaviour**	PB1 PB2 PB3 PB4	0.862 0.860 0.847 0.890	0.888	0.922	0.748
**Perceived Behavioural Control**	PBC1 PBC2 PBC3 PBC4 PBC5	0.876 0.842 0.787 0.829 0.868	0.896	0.923	0.707
**Purchase Intention**	PI1 PI2 PI3 PI4	0.779 0.820 0.808 0.848	0.830	0.887	0.663
**Product Knowledge**	PKN1 PKN2 PKN3 PKN4 PKN5	0.749 0.847 0.865 0.890 0.866	0.899	0.925	0.714
**Subjective Norms**	SN1 SN2 SN3 SN4 SN5 SN6	0.832 0.806 0.836 0.841 0.823 0.811	0.906	0.927	0.680

**Source:** Author’s Data analysis.

## Results

### Factors affecting consumers' purchase intention, and purchase behaviour: A PLS-SEM Model

One of the more advanced modelling techniques, partial least squares structural equation modelling (PLS-SEM), is frequently required to properly comprehend the intricate and multifaceted elements influencing consumers' purchasing intention and conduct. Using this technique, researchers can investigate the intricate relationships that exist between several factors, such as perceived value, product attributes, social impact and brand image. With PLS-SEM, researchers can determine the direct and indirect effects of these attributes on customers' actual purchasing behaviour and intentions. These findings provide significant insights that organizations can use to improve customer satisfaction and marketing tactics. These models provide an advanced understanding of the dynamic mechanisms affecting consumer decisions in the modern economy. The model's initial output is called the measurement model.

### Measurement Model

Using SPPS 23, an EFA was carried out to lower the construct's variable count prior to SEM analysis. The author used varimax rotation and set the latent root criterion to 1.0 for including factors. The author also set a cut-off of 0.70 for factor inclusion and factor loading. Table 2 confirms that all factor loading values in the proposed model were above the recommended minimum of 0.7, meaning the items in the construct had high loading. These results show that the model was a suitable explanation for the dimensionality ( Vinodkumar & Bhasi, 2009; Shook *et al*., 2004). All the constructs' Cronbach Alpha levels were significant (>0.70), indicating that the model is reliable ( Shook *et al.*, 2004; Chin *et al*., 2018). According to Table 2, it is confirmed that all six factors have sufficient convergent validity since each latent variable has a CR above 0.70 and an AVE above 0.50, which surpasses the required cut-off criteria for both CR and AVE values ( Chin *et al*., 2018; Chin *et al*., 2018) **.** In each case, the AVE of each latent variable was larger than the squared correlation coefficient that corresponded with it. This demonstrates that the constructs are distinct and explained well by their measurement scales. As a result, authors have ensured convergence and discriminant validity, which is suitable for our measurement scale ( Fornell & Larcker, 1981; Yu & Lee, 2019).

*Discriminant validity Analysis*

The Discriminant validity of the EC, PBC, PKN, and SN in relation to PB and PI in the measurement scale implies that each construct is perceived differently and does not have high correlations. This means that respondents react differently to each construct. Table 3 shows values less than 0.8, which indicates that the HTMT criterion supports the existence of discriminant validity in the responses received for the measurement scale. Furthermore, the Discriminant validity of the present measurement scale, which measures the PB and PI of consumers for green cosmetic products, is evaluated using the Fornell Larcker criteria. This involves comparing the square root of the AVE of each construct with its correlation with all other constructs in the measurement model. The square root of the AVE is displayed on the main diagonal of the table, and the correlation between the constructs is displayed in the remaining cells. The result reveals that the square root of the AVE of individual constructs is more than its relationship with the other construct in the present measurement model. This confirms the presence of discriminant validity of the present measurement model.

**Table 3.** HTMT (Heterotrait – Monotrait ratio).

	EC	PBC	PKN	PB	PI	SN
**EC**						
**PBC**	0.729					
**PKN**	0.514	0.505				
**PB**	0.535	0.607	0.657			
**PI**	0.445	0.557	0.541	0.749		
**SN**	0.526	0.532	0.559	0.778	0.599	

**Note:** EC- Environment Concern, PBC- Perceived behavioural control, PKN- Product Knowledge, PB- Purchase behaviour, PI- Purchase Intention, SN- Subjective Norms.

**Source:** Author’s Compilation.

### Factors affecting consumers' purchase intention and purchase behaviour across gender: Multi-Group Analysis

To assess variations based on the gender of the consumers, a Multi-Group Analysis was conducted. A non-parametric test was also performed to understand and study the changes in the relationship between the models specifically based on the gender features of the respondents. Table 4 presents the path values of two groups of male and female consumers, along with the differences within the group and their respective p-values, as suggested by ( Henseler & Ringle, 2009). The PMGA (Private Multi-Group Aggregation) displays the significance of group differences from PLS-SEM using p-values, shown in Table 5.

**Table 4.** Fornell- Lacker Criterion.

	EC	PBC	PKN	PB	PI	SN
**EC**	**0.826**					
**PBC**	0.642	**0.841**				
**PKN**	0.459	0.453	**0.845**			
**PB**	0.478	0.548	0.588	**0.865**		
**PI**	0.384	0.490	0.472	0.645	**0.814**	
**SN**	0.475	0.486	0.508	0.704	0.525	**0.825**

**Note:** EC- Environment Concern, PBC- Perceived behavioural control, PKN- Product Knowledge, PB- Purchase behaviour, PI- Purchase Intention, SN- Subjective Norms.

**Source:** Author’s Compilation.

**Table 5.** Multi-Group Analysis.

	β Value (F)	β Value (M)	Mean (F)	Mean (M)	STDEV (F)	STDEV (M)	t value (F)	t value (M)	p value (F)	p value (M)
**EC-PI**	0.021	-0.118	0.02	-0.114	0.111	0.089	0.192	1.323	0.848	0.187
**PBC - PB**	0.226	0.326	0.233	0.318	0.115	0.095	1.954	3.437	0.051	0.001
**PBC - PI**	0.432	0.392	0.431	0.39	0.124	0.103	3.473	3.81	0.001	0.00
**PKN - EC**	0.521	0.553	0.52	0.554	0.082	0.063	6.348	8.73	0.00	0.00
**PI - PB**	0.575	0.59	0.569	0.596	0.103	0.083	5.564	7.138	0.00	0.00
**SN - PI**	0.407	0.433	0.415	0.435	0.109	0.087	3.742	4.949	0.00	0.00

**Note**: EC- Environment Concern, PBC- Perceived behavioural control, PKN- Product Knowledge, PB- Purchase behaviour, PI- Purchase Intention, SN- Subjective Norms, STDEV- standard deviation, F- Female, M- Male.

**Source:** Author’s Compilation.

Based on the sample collected from the male and female respondents, it was seen in the results that the two groups had a notable difference in the connection between the perceived behavioural control and purchase intentions of female and male consumers. Additionally, there is a significant difference in the purchase behaviour and intentions between female and male respondents. However, gender did not impact the correlation between environment concerns, subjective norms, perceived behaviour control and product knowledge to purchase green cosmetic products. Table 6 displays the results.

**Table 6.** Group Analysis results for gender.

	Male		Female		Differences		
	β -Value	p-value	β -Value	p-value	β -Value	p-value	Decision
**EC-PI**	-0.118	0.187	0.021	0.848	-0.094	0.169	Not Significant
**PBC - PB**	0.326	0.001	0.226	0.051	0.113	0.147	Not significant
**PBC - PI**	0.392	0.00	0.432	0.001	0.133	0.015	Sig. Difference
**PKN - EC**	0.553	0.00	0.521	0.00	0.302	0.141	Not Significant
**PI - PB**	0.59	0.00	0.575	0.00	0.227	0.030	Sig. Difference
**SN - PI**	0.433	0.00	0.407	0.00	0.026	0.138	Not Significant

**Note**: EC- Environment Concern, PBC- Perceived behavioural control, PKN- Product Knowledge, PB- Purchase behaviour, PI- Purchase Intention, SN- Subjective Norms, STDEV- standard deviation, F- Female, M- Male.

**Source:** Author’s Compilation.

**Figure 2.**
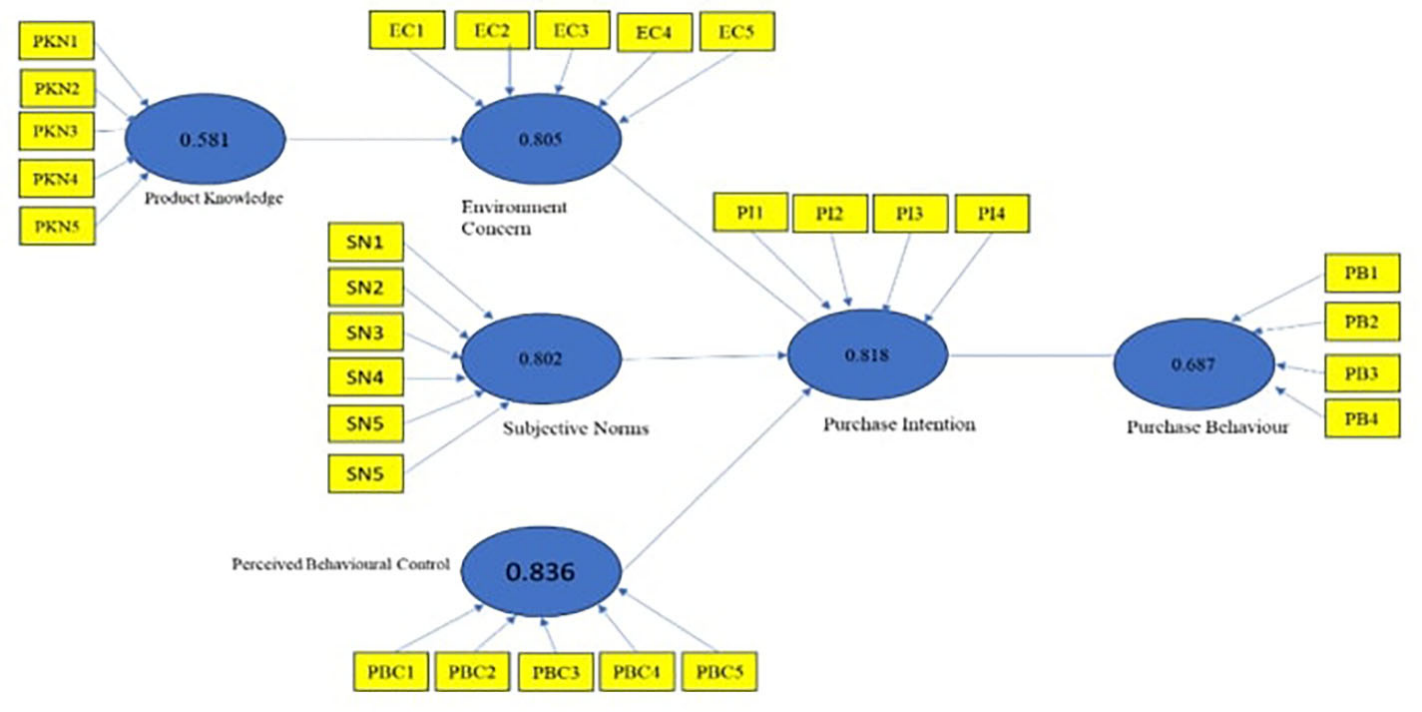
Structural model constructed using smart PLS. **Source:** Author’s Data analysis.

The study's first objective involved identifying variables from the systematic literature review related to the constructs present in the TPB. All the articles referred to in the study were from Scopus-listed journals, web of Science, Research Gate, Emerald Insight and Google Scholar and a gap was identified among them. As part of the second objective, we utilized the TPB and SEM ( Figure 2) to understand the connection between the variables that affected the purchase intentions and purchasing behaviour of consumers toward green cosmetic products specific to the respondents' gender. SEM was used to test relevant hypotheses in Smart PLS 3.0 to determine if the relationships between variables were significant. The final model to understand the purchase intentions of male and female consumers was created based on hypothesis testing. Environment Concerns did not significantly impact purchase intention, while product knowledge, subjective norms, and perceived behavioural control positively impacted purchase intention for green cosmetics. The study found that knowledge about a green product directly impacted a person's environmental concern, but it did not influence their intention to purchase green cosmetics. However, it was found that having an intention to purchase green cosmetic products had a significant and positive impact on the actual purchase behaviour of the individuals.

About the third objective, it can be noted that perceived behavioural control and subjective norms have a positive and significant influence on the purchase behaviour towards green cosmetic products for both males and females. However, it was found that environmental concerns do not significantly impact consumers' purchase intentions toward green cosmetic products. Therefore, it can be inferred that there are no substantial differences between males and females concerning their purchase intentions and the factors that influence them. The last objective was to develop the purchase intentions of the consumers and their purchase behaviour model towards green cosmetic products. Modelling details are presented in Figure 2, and Table 7 explains the relationship between variables.

**Table 7.** Hypothesis Results.

S.No	Dependent Construct	Hypothesis	p and β value	Inference
1	**PKN - EC** 0.141	H _1_ = Consumers product knowledge and its influence on environmental concern is gender neutral	Β=0.302 P=0.141	Insignificant
2	**PBC - PB**	H _3_ Perceived Behavioural Control (PBC) and its influence on purchase behaviour is gender neutral	β = 0.113 P = 0.147	Insignificant
3	**EC-PI**	H _5_ = consumer Environmental concern and its influence on purchase intention is gender neutral	Β= -0.094 P=0.169	Insignificant.
4	**PI - PB**	H4: Consumer purchase intention and its effect on purchase behaviour are gender neutral	β = 0.227 P = 0.030	significant
5	**SN - PI**	H5 = Subjective norm and its influence on purchase intention of cosmetics are gender neutral	β = 0.026 P = 0.138	Insignificant.

**Source:** Author’s Compilation.

Research reveals several significant elements that impact the purchasing decisions of both male and female consumers regarding green cosmetic goods. Particularly for male consumers, it was discovered that their opinions on green cosmetics, product knowledge, and subjective standards were all favourably correlated with their purchase intentions. Remarkably, their inclinations to purchase were not significantly influenced positively by environmental concerns.

Our study found that female customers' inclinations to buy green cosmetics were positively connected with attitudes, subjective norms and product knowledge. Similar to their male colleagues, their purchasing intentions were unaffected directly by environmental concerns. However, product knowledge was crucial in predicting how they felt about environmental issues, similar to the findings for male consumers. These studies demonstrate the significance of factors such as attitudes, subjective norms, and product knowledge in affecting the purchase intentions of both male and female consumers. It is argued that societal norms and product-related factors have a substantial impact even when they do not directly influence purchasing decisions. When it came to green cosmetics, there were no appreciable differences between male and female consumers' intentions or buying habits.

## Discussions

The research revealed no statistically significant variations in India's purchase behaviour or intentions with regard to eco-friendly cosmetics based on gender. According to the study, both male and female customers' purchase intentions for green cosmetics are positively impacted by subjective standards. Moreover, a significant element influencing how customers behave when making purchases is their intention to buy.

The factors that influence purchase intentions tend to shape the purchasing behaviour of male and female consumers toward green cosmetic products in India ( Vinod Kumar & Bhasi, 2009). Additionally, the study found that perceived behavioural control also positively influences consumers' purchase behaviour towards green cosmetic products. According to the Theory of Planned Behavior, perceived behavioural control affects both consumers' purchase behaviour and purchase intention.

It can be concluded that PBC (Perceived Behavioral Control) significantly impacted purchase behavior, both directly and indirectly. However, product knowledge of green cosmetic products only impacts consumers' environmental concerns without affecting their purchase intentions for these products. Therefore, we can conclude that knowledge regarding green products can raise consumers' awareness of environmental issues, but this knowledge does not influence their purchase intentions. So we could say that among all the variables, environmental concern was the only factor that was not influencing the purchase intentions and purchase behaviour of male and female consumers toward green cosmetic products.

We have found that consumer behavior is a result of a combination of factors. Some belong to the wider consumer environment, such as culture, social context, and family, which comes under subjective norms. Others belong to specific individuals, such as perceived behavioral control, product knowledge, and environmental concern.

The results of the study can provide guidance to organizations regarding the factors that affect consumer behavior and their intention to buy environmentally friendly cosmetic products. As a result, the company can implement an effective green strategy that enables it to achieve better segmentation and positioning compared to conventional cosmetic products.

### Theoretical Implication

Significant theoretical ramifications flow from the study's findings, which provide a gender-neutral perspective on the dynamics of consumer behaviour and purchase intentions for green cosmetic items in North India. By applying the Theory of Planned Behaviour (TPB) and Structural Equation Modelling (SEM), the research elucidates the complex interrelationships among diverse factors that impact purchasing intentions, with a particular focus on gender-specific subtleties. Subjective norms, perceived behavioural control, product knowledge, and environmental concerns did not significantly influence purchase intention; nevertheless, they were key factors. Notably, product knowledge did not directly influence purchase intentions, even though it favourably increased environmental concerns. This highlights the intricate relationship between the creation of intentions and knowledge acquisition. The study also shows that intention to buy green cosmetics influences actual purchase behaviour in a considerable way, closing the gap between intention and behaviour. These results highlight intention's critical role in behaviour prediction and shed light on the varying effects of variables on buying intentions across genders, which helps to improve theoretical frameworks like TPB.

### Managerial Implication

From a management perspective, the report provides useful information to businesses involved in North India's green cosmetics industry. Through the identification of the variables impacting consumer behaviour and purchase intents, companies can create more specialised marketing campaigns. The significant positive influence of subjective standards and perceived behavioural control on purchasing decisions suggests the significance of social influences and personal empowerment. The study also demonstrates that purchasing intentions are not significantly influenced by environmental concerns. This means that businesses should focus on enhancing consumer awareness of their products and fostering favourable perceptions of green cosmetics to boost demand. This demonstrates how critical it is to start public awareness campaigns and educational initiatives to raise consumer understanding of environmental challenges and the benefits of eco-friendly products. By including these insights in their strategy, companies can strengthen their competitiveness and market positioning in the expanding green cosmetics industry. This will help promote environmentally responsible behaviour and sustainable consumption patterns.

## Conclusions

After conducting research and discussing the findings, the study concludes that environmental concern is not significantly correlated with the purchase intentions of male and female consumers in India regarding green cosmetic products. This suggests that even if a consumer has a high level of environmental awareness, they may not necessarily feel a strong desire to purchase green cosmetic products as they may not believe such an act would significantly contribute to preserving the environment.

It has been observed that there is a significant correlation between consumers' knowledge about a product and their concern for the environment. In other words, if consumers possess sufficient knowledge about a product, it will undoubtedly affect their perception of the product's features that help in environmental safety. But ultimately it does not influence their decision to purchase the product.

The study also discovered that subjective norms significantly impact the purchase intentions of both male and female consumers when it comes to green cosmetic products. This means that individuals who receive positive feedback from their close friends or relatives are likelier to have a favourable attitude towards such products. Additionally, social media advertisements play a role in encouraging potential buyers to see the benefits of these cosmetic products, eventually leading to their purchase. It's important to note that a person's decision to use and purchase green cosmetics should come from within rather than being influenced by others if we want it to continue for a longer period. Research shows that perceived behavioural control, as a dependent variable, has a significant positive relationship with purchase intentions and purchase behaviour of both male and female Indian consumers towards green cosmetic products. With this, we can understand that if someone gets the right opportunity and a sufficient amount of money and time, then it will drive the consumer toward the green cosmetic product. This will help create a need, desire and demand for the consumer for the green cosmetic product in India.

Based on the research findings, it is recommended that companies should have a strong grip on marketing strategies to attract more male consumers towards green cosmetic products. Presently, limited advertisements are targeting male consumers for cosmetic products, which are easily noticeable. Companies should also try to be more responsible toward the environment and start producing cosmetic products which are environment friendly as well as good for the skin of the consumers. For future research, the author wants to suggest that the transgender community should also be explored. We need to see if these factors also influence the transgender in the same way, and we also need to explore the needs of the transgender in terms of cosmetic products and their purchase behaviour toward them.

### Limitations and future direction of the study

**Limitations of the study**

While this study provided valuable insights into the dynamics of consumer behaviour and purchase intentions towards green cosmetic products in North India, several limitations should be acknowledged. Firstly, the research focused solely on North India, limiting the generalizability of findings to other regions. Additionally, the study employed a cross-sectional design, which may not capture changes in consumer behaviour over time. In addition, the use of self-reported data raises the possibility of social desirability bias, which could affect response accuracy. Future research should consider longitudinal methodologies and a broader geographic scope to improve the generalizability of outcomes.

### Future directions

Further research should investigate several paths to gain a deeper understanding of consumer behaviour regarding green cosmetic goods, building on the findings of this study. First, it might be insightful to look into how cultural influences affect how consumers feel and act toward eco-friendly items. A fuller understanding of the sustainability trends in the cosmetics business could be obtained by using longitudinal research to monitor changes in consumer preferences and behaviours over time. Also, qualitative research techniques like focus groups and interviews may offer a more in-depth understanding of the underlying motives influencing consumer decisions. Future studies can deepen our understanding of how consumers behave toward eco-friendly cosmetics and provide guidance for sustainable marketing and product development tactics by exploring these pathways.

## Ethical statement

This research was conducted under the guidelines of the **Research Ethics Board** (REB) of **Uttaranchal University**. The Research Ethics Board was approved on March 4, 2023, and the approval number is UU/DRI/EC/2023/003.

## Informed consent

All the participants involved in the study have given consent. A self-explanatory written statement was attached to the questionnaire for the participants, and a similar questionnaire has been submitted to the university research board (REB).

## Data Availability

The underlying data related to the paper is available in Figshare with the following citations and DOI: Figshare: Singh, neha (2024). research paper data set.xls. Figshare. Dataset. https://doi.org/10.6084/m9.figshare.25498282.v1 Understanding the Dynamics of Consumer Behaviour and Purchase Intentions for Green Cosmetic Products in North India: A Gender-Neutral Approach © 2024 by Neha Singh is licensed under CC BY 4.0. To view a copy of this licence, visit https://creativecommons.org/licenses/by/4.0/. All the data was collected from the respondents by filling out the questionnaire and can be made public in SPSS file format if required. Data can be provided by the author if it is very important, as the same data has been used for thesis analysis.
