# Supplementary material for: Understanding the Dynamics of consumer behaviour and purchase intentions for Green Cosmetic Products in North India: A Gender-Neutral Approach
Source: F1000Res. 2025 Jan 29;13:611. Originally published 2024 Jun 10. [Version 2] doi: 10.12688/f1000research.151629.2 (PMC11799759; doi:10.12688/f1000research.151629.2)
Supplement: Peer review history

# Reviewer report: Reviewer response for version 2

**DOI:** 10.5256/f1000research.177375.r363362

**Published:** 2025-02-05

**Authors:** Ishani Patharia Chopra

**Affiliations:** 1 Department of Commerce, Bhagat Phool Singh Mahila Vishwavidyalaya, Sonipat, Haryana, India

The improved version of the article is more readable, compares the context to existing research on this domain, and precisely presents the contributions to the body of knowledge.

 Congratulations to the authors for their contribution and indexing.

Is the work clearly and accurately presented and does it cite the current literature?

Partly

If applicable, is the statistical analysis and its interpretation appropriate?

Partly

Are all the source data underlying the results available to ensure full reproducibility?

Yes

Is the study design appropriate and is the work technically sound?

Yes

Are the conclusions drawn adequately supported by the results?

Yes

Are sufficient details of methods and analysis provided to allow replication by others?

Yes

Reviewer Expertise:

Marketing, Consumer /behaviour, Sustainability

I confirm that I have read this submission and believe that I have an appropriate level of expertise to confirm that it is of an acceptable scientific standard.

# Reviewer report: Reviewer response for version 1

**DOI:** 10.5256/f1000research.166289.r334012

**Published:** 2025-01-07

**Authors:** Adilla Anggraeni

**Affiliations:** 1 Bina Nusantara University, Jakarta, Indonesia

The study is interesting and provides insights into green purchase intention. However, it is recommended to provide more details into the justification on the respondent criteria. 

 Should the respondents be customers/consumers of a particular green cosmetic brand? Even though the study was conducted in North India, the study may not have to mention it unless it can provide differences/comparisons of green consumption behaviours of North Indians vs South Indians.

Is the work clearly and accurately presented and does it cite the current literature?

Yes

If applicable, is the statistical analysis and its interpretation appropriate?

Yes

Are all the source data underlying the results available to ensure full reproducibility?

Yes

Is the study design appropriate and is the work technically sound?

Yes

Are the conclusions drawn adequately supported by the results?

Yes

Are sufficient details of methods and analysis provided to allow replication by others?

Partly

Reviewer Expertise:

Consumer Behaviour, Retail, Social Marketing

I confirm that I have read this submission and believe that I have an appropriate level of expertise to confirm that it is of an acceptable scientific standard.

# Response

**Published:** 2025-01-22

**Authors:** Neha Singh

The language of the article has been clarified and further explanations of the test will be provided within the article.

# Reviewer report: Reviewer response for version 1

**DOI:** 10.5256/f1000research.166289.r295409

**Published:** 2024-07-11

**Authors:** Ishani Patharia Chopra

**Affiliations:** 1 Department of Commerce, Bhagat Phool Singh Mahila Vishwavidyalaya, Sonipat, Haryana, India

The paper is well designed and adds to the green purchase literature but there are some issues that can be resolved:

 1. An English editing would be helpful to improve presentation.

 2. Many of the seminal papers using similar model and factors are missing. For example:

     (i) Patharia, I., Rastogi, S., Vinayek, R., & Malik, S. (2020). A fresh look at environment friendly customer's profile: Evidence from India. *International journal of economics and business research*, *20*(3), 310-321.

    (ii)   Patharia, I., Rastogi, S., Vinayek, R. (2022). Determining Green Purchase Behaviour Towards Electronic Products in an Emerging Economy: Theory and Evidence. In: Nguyen, N., Nguyen, H.V., D'Souza, C., Strong, C. (eds) Environmental Sustainability in Emerging Markets. Approaches to Global Sustainability, Markets, and Governance. Springer, Singapore. https://doi.org/10.1007/978-981-19-2408-8_4

 (iii) Sharma, K., Aswal, C., & Paul, J. (2023). Factors affecting green purchase behavior: A systematic literature review. *Business Strategy and the Environment*, *32*(4), 2078-2092.

 (iv) Yusoff, N., Alias, M., & Ismail, N. (2023). Drivers of green purchasing behaviour: a systematic review and a research agenda. *F1000Research*, *12*.

 Therefore, the authors must include them in the manuscript.

 3. The format of citations is inconsistent which needs to checked and improved.

 4. The hypotheses are poorly framed and the hypothese for main effects are missing as the current hypotheses focus on moderation effects and not the direct effects.

 5. There is no literature review provided for moderation effects.

 6. The sequencing of theoretical framework and literature review are incorrect.

 7. The discussion on novelty of contributions of this paper need to be elaborated.

 8. Please provide the statements used for measuring the constructs.

 9. Various model fit indices such as R square, Q square and F suare have not been reported.

 10. The managerial implications need to be elaborated especially around moderation effect.

Is the work clearly and accurately presented and does it cite the current literature?

Partly

If applicable, is the statistical analysis and its interpretation appropriate?

Partly

Are all the source data underlying the results available to ensure full reproducibility?

Yes

Is the study design appropriate and is the work technically sound?

Yes

Are the conclusions drawn adequately supported by the results?

Yes

Are sufficient details of methods and analysis provided to allow replication by others?

Yes

Reviewer Expertise:

Marketing, Consumer /behaviour, Sustainability

I confirm that I have read this submission and believe that I have an appropriate level of expertise to confirm that it is of an acceptable scientific standard, however I have significant reservations, as outlined above.

## References

[1] : A fresh look at environment friendly customer's profile: Evidence from India. *International journal of economics and business research* .2020-Volume 20(3);

[2] : Mitigating Harms of Social Media for Adolescent Body Image and Eating Disorders: A Review. *Psychol Res Behav Manag* .2024;17: 10.2147/PRBM.S410600 2587-2601 10.2147/PRBM.S410600 38978847 PMC11229793

[3] : Factors affecting green purchase behavior: A systematic literature review. *Business Strategy and the Environment* .2023-Volume 32(4),2078-2092;

[4] : Drivers of green purchasing behaviour: a systematic review and a research agenda. *F1000Researxh* .2023;10.12688/f1000research.140765.1PMC1077463338196406

# Response

**Published:** 2025-01-22

**Authors:** Neha Singh

As per the reviewer's feedback, I will incorporate the references provided to enhance the article's depth and credibility. The language of the article will be revised for clarity and precision. Additionally, the statements used to test the constructs will be included in the article to ensure comprehensiveness and alignment with the reviewer's suggestions. More clarity will be provided for the test used in the article.
